# RNA-Seq reveals the key pathways and genes involved in the light-regulated flavonoids biosynthesis in mango (*Mangifera indica* L.) peel

**DOI:** 10.3389/fpls.2022.1119384

**Published:** 2023-01-18

**Authors:** Minjie Qian, Hongxia Wu, Chengkun Yang, Wencan Zhu, Bin Shi, Bin Zheng, Songbiao Wang, Kaibing Zhou, Aiping Gao

**Affiliations:** ^1^ Sanya Nanfan Research Institute of Hainan University, Sanya, China; ^2^ Ministry of Agriculture Key Laboratory of Tropical Fruit Biology, South Subtropical Crops Research Institute, Chinese Academy of Tropical Agricultural Sciences, Zhanjiang, China; ^3^ Key Laboratory of Quality Regulation of Tropical Horticultural Crop in Hainan Province, School of Horticulture, Hainan University, Haikou, China; ^4^ Tropical Crops Genetic Resources Institute, Chinese Academy of Tropical Agricultural Sciences & Ministry of Agriculture Key Laboratory of Crop Gene Resources and Germplasm Enhancement in Southern China, Haikou, China

**Keywords:** mango, flavonoids, bagging treatment, RNA-seq, transcription factor

## Abstract

**Introduction:**

Flavonoids are important water soluble secondary metabolites in plants, and light is one of the most essential environmental factors regulating flavonoids biosynthesis. In the previous study, we found bagging treatment significantly inhibited the accumulation of flavonols and anthocyanins but promoted the proanthocyanidins accumulation in the fruit peel of mango (*Mangifera indica* L.) cultivar ‘Sensation’, while the relevant molecular mechanism is still unknown.

**Methods:**

In this study, RNA-seq was conducted to identify the key pathways and genes involved in the light-regulated flavonoids biosynthesis in mango peel.

**Results:**

By weighted gene co-expression network analysis (WGCNA), 16 flavonoids biosynthetic genes were crucial for different flavonoids compositions biosynthesis under bagging treatment in mango. The higher expression level of *LAR* (*mango026327*) in bagged samples might be the reason why light inhibits proanthocyanidins accumulation in mango peel. The reported *MYB* positively regulating anthocyanins biosynthesis in mango, *MiMYB1*, has also been identified by WGCNA in this study. Apart from MYB and bHLH, ERF, WRKY and bZIP were the three most important transcription factors (TFs) involved in the light-regulated flavonoids biosynthesis in mango, with both activators and repressors. Surprisingly, two *HY5 *transcripts, which are usually induced by light, showed higher expression level in bagged samples.

**Discussion:**

Our results provide new insights of the regulatory effect of light on the flavonoids biosynthesis in mango fruit peel.

## Introduction

Flavonoids are a large group of water soluble secondary metabolites that are widely distributed in plants. Flavonoids play a diversity of roles in extant plants such as floral coloration for attracting pollinators (Schaefer et al., 2004), protection against biotic and abiotic stresses including UV irradiation (Qian et al., 2021), nitrogen deficiency (Lea et al., 2007), drought (Ma et al., 2014), cold (Sudheeran et al., 2018), fungal pathogens (Barcelóet al., 2017 ), and pest (Casas et al., 2016). In addition, flavonoids are also beneficial for human health due to their antioxidant activities against free radicals, subsequently reducing the risk of chronic diseases, especially cancer (Chen and Chen, 2013). Flavonols, anthocyanins, and proanthocyanidins (PAs) are the three main flavonoid subgroups in various higher plant species (Williams and Grayer, 2004).

Flavonoids biosynthesis starts with general phenylpropanoid pathway (Winkel-Shirley, 2001). After being catalyzed by Phenylalanine ammonia-lyase (PAL), cinnamic acid 4-hydroxylase (C4H), and 4-coumarate:CoA ligase (4CL), phenylalanine is consequently converted to 4-coumaroyl-CoA. The rate limiting entry into the flavonoid pathway is controlled by chalcone synthase (CHS), which catalyzes the condensation of three molecules of malonyl-CoA with 4-coumaroyl-CoA into a chalcone (Zhang et al., 2017). Flavonols, anthocyanins, and proanthocyanidins are three branches derived from flavonoids pathway, which share the same enzymes including CHS, chalcone isomerase (CHI), flavanone 3-hydroxylase (F3H), and flavonoid 3’-hydroxylase (F3’H) to form dihydroflavonols. Dihydroflavonols are further converted to flavonols by flavonol synthase (FLS) or to leucoanthocyanidins by dihydroflavonol reductase (DFR). *Via* anthocyanidin synthase (ANS) and UDP-glucose: flavonoid 3-*O*-glucosyltransferase (UFGT), leucoanthocyanidins are firstly converted to anthocyanidins, and consequently to anthocyanins. PAs are synthesized from leucocyanidins by leucoanthocyanidin reductase (LAR) or from anthocyanidins by anthocyanidin reductase (ANR). The transcriptional regulation of flavonoids biosynthesis is through the MYB-bHLH-WD40 complex, with the essential role of MYB transcription factor (TF) (Broun, 2005). In Arabidopsis, there are 125 R2R3-MYB TFs, which can be divided into 25 subgroups, and the 5^th^, 6^th^ and 7^th^ subgroups are involved in the biosynthesis of proanthocyanidins, anthocyanins, and flavonols, respectively (Stracke et al., 2001; Dubos et al., 2010).

Flavonoids biosynthesis in fruit is affected by environmental factors, and light is one of the most important factors. Numerous fruit bagging and shading experiments have shown light conditions play a key role in regulating flavonoids accumulation in grape berry (Cortell and Kennedy, 2006), apple (Ryu et al., 2022), pear (Qian et al., 2013; Sun et al., 2014), litchi (Ma A. et al., 2021), cucumber (Qian et al., 2021), and mango (Karanjalker et al., 2018; Kanzaki et al., 2020). Light signal pathway key proteins COP1 and HY5 participate in the light-induced flavonoids biosynthesis. COP1, an ubiquitin E3 ligase, is located in nucleus in darkness to mediate the ubiquitination and degradation of MYB1 to repress anthocyanin accumulation, while in light, nuclear depletion of the COP1 protein leads to the MYB1 accumulation and subsequent fruit coloration in apple (Li et al., 2012). HY5 is a light-responsive TF, which could bind to the G-box or ACE-box in the promoter region of target genes to activate the expression of flavonoids biosynthesis related genes including *CHS*, *ANS*, *FLS*, and *MYB*, to promote the light-induced flavonoids accumulation in apple (Henry-Kirk et al., 2018), pear (Tao et al., 2018), and grape (Loyola et al., 2016). Other TFs such as NAC (Morishita et al., 2009), WRKY (Wang et al., 2018), ERF (Ni et al., 2019; Ni et al., 2021; Zhao et al., 2021), and BBX (Bai et al., 2019a; Bai et al., 2019b; Li et al., 2021) have also been reported to participate in flavonoids biosynthesis.

Mango (*Mangifera indica* L.) is the fifth most produced fruit crop worldwide (http://www.fao.org/faostat/), which is widely cultivated in tropical and subtropical areas in the world. So far, the molecular mechanism of flavonoids biosynthesis in mango is mainly focusing on the expression changes of flavonoids biosynthetic genes and MBW complex by different treatments or in different cultivars (Hoang et al., 2015; Kanzaki et al., 2019; Kanzaki et al., 2020), while how these genes are regulated by the up-stream TFs is still unknown. In the previous study, we found bagging treatment significantly decreased the anthocyanins and flavonols but surprisingly increased the proanthocyanidins accumulation in the fruit peel of red mango cultivar ‘Sensation’ (Shi et al., 2021), which was very different from the other studies since light generally promotes all flavonoids compounds accumulation including flavonols, anthocyanins and proanthocyanidins (Cortell and Kennedy, 2006; Li et al., 2021). We only analyzed anthocyanin biosynthetic and regulatory gene expression in the previous study (Shi et al., 2021). Therefore, it is very interesting to further reveal the molecular mechanism of light-promoted anthocyanins and falvonols but -repressed proanthocyanidins accumulation in mango.

In this study, samples collected in the previous study (Shi et al., 2021), i.e. bagged fruit peel and natural light grown fruit peel (control) of red mango cultivar ‘Sensation’ sampled at three developmental stages, i.e. 50 days after full bloom (DAFB), 80 DAFB, and 120 DAFB were used for RNA sequencing (RNA-Seq). Weighted gene co-expression network analysis (WGCNA) was used to identify light-responsive genes especially regulatory genes which could encode TFs to contribute to the process of light-induced anthocyanins and falvonols but light-inhibited proanthocyanidins biosynthesis in mango. This study will enrich our knowledge regarding the regulation of light on flavonoids biosynthesis in fruit.

## Materials and methods

### Plant materials and treatments

The fruits of ‘Sensation’ mango were obtained from the mango field genebank of South Subtropical Crops Research Institute (SSCRI) in Zhanjiang, China. Three trees were selected and 50 fruits per tree were bagged with double layers yellow black paper bags (Qingdao Kobayashi Co., Ltd., Qingdao, China) at 20 days after full bloom (DAFB) to block out all the light regarded as bagging treatment. The rest fruits exposing to sunlight were regarded as control. Ten fruits of bag-treated and control per tree were harvest at 50, 80, and 120 DAFB, respectively. After measuring the fruit color index by a portable colorimeter (LS170, Shenzhen Linshang Technology Co.,Ltd., Shenzhen, China), fruit peels were collected in liquid nitrogen and stored at -80 °.

### RNA extraction and sequencing

Total RNA was extracted by a RNA prep pure plant kit (Tiangen, DP441, Beijing, China). After being enriched and fragmented, mRNA was reverse-transcribed to cDNA. The cDNA underwent purification, end repair, and A-tail addition, and was subsequently ligated to the adapters. Approximately 200 bp cDNA was screened by AMPure XP beads, and enriched cDNA by PCR amplification was used for library construction. Two end RNA sequencing (paired-end) was based on the Illumina sequencing platform by Metware Biotechnology Co., Ltd. (Wuhan, China). Clean reads were obtained after the removal of low quality data from the raw reads by Fastp software (https://github.com/OpenGene/fastp), and subsequently mapped to the mango reference genome (BIG Genome Sequence Archive database, accession number: PRJCA002248) using TopHat (Trapnell et al., 2012). Transcripts were assembled from the reads by Cufflinks and Fragments Per Kilobase of transcript per million fragments mapped (FPKM) was used to calculate the gene expression. DESeq R package (1.10.1) was used to analyze the differential expression between two groups. Genes with a significant *p*-value < 0.05 and |log_2_FoldChange| > 1 were regarded as differentially expressed genes. The cluster analysis was conducted by the Mfuzz package in R. The raw data of RNA-seq was submitted to NCBI with the following ID number: PRJNA905802.

### cDNA synthesis and quantitative real-time PCR

cDNA was synthesized from 1 µg of total RNA by HiScript IIQ RT SuperMix (Vazyme, R223-01, Nanjing, China) according to the manufacturer’s instructions. Quantitative real-time PCR (Q-PCR) was conducted as described by Shi et al. All the primers for Q-PCR were designed by primer3 (https://bioinfo.ut.ee/primer3-0.4.0/) and listed in Supplementary File S1. Gene expression was calculated by the 2^-ΔΔCt^ method, and mango *actin* gene was used for normalization.

### WGCNA analysis

The WGCNA analysis was conducted by WGCNA (v1.29) package in R (Langfelder and Horvath, 2008). The concentration of flavonols, anthocyanins, and proanthocyanidins in the peel of bagged or unbagged mango fruits during different developmental stages, as well as all the expressed genes detected by RNA-seq (28851 genes), were used for WGCNA. The modules were built by the automatic network construction function ‘blockwise’. The soft power, minModuleSize, and mergeCutHeight were set to 4, 30, and 0.25, respectively. The eigengene value was calculated for each module and used for testing the association with each sample or traits. The soft thresholding was used to keep the continuous nature of the data set and prevent setting an arbitrary correlation score cutoff. Candidate genes from ‘purple’, ‘darkgreen’, ‘grey60’, ‘orange’ and ‘midnightblue’ were selected by thresholding at a value of 0.80. Kyoto Encyclopedia of Genes and Genomes (KEGG, http://www.genome.jp/kegg) database were used for the functional annotation of genes.

### Statistical analysis

Data were presented as mean value ± standard deviation. Experimental Data were subjected to a Student’s *t*-test using SPSS 27.0 (SPSS, Chicago, IL, USA). Probability values of <0.05 were considered statistically significant, and marked with one asterisk (*). Probability values of <0.01 were considered highly statistically significant, and marked with two asterisks (**).

## Results

### Fruit color analysis

**Figure 1:**
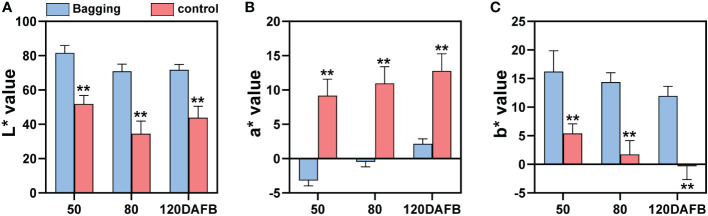
Changes of fruit color index L* **(A)**, a* **(B)**, and b* **(C)** of bagged and control (non-bagged) ‘Sensation’ mango fruits during different developmental stages. Each value represents the mean ± standard deviation of three biological replicates. * indicates significant difference (*p*-value < 0.05). ** indicates very significant difference (*p*-value < 0.01), as determined by Student’s *t*-test.

Fruit color index L*, a*, and b* represent lightness, red (+) or green (-), and yellow (+) or blue (-), respectively. Non-bagged ‘Sensation’ fruits were dark-red colored during all developmental stages, so they showed relatively low L*, high a*, and low b* values (**Figure 1**). In contrast, bagged ‘Sensation’ fruits exhibited light white-yellow coloration, quantified as high L*, low a*, and high b* values (**Figure 1**).

### Library construction and transcriptome sequencing

The peel of natural light grown fruits and bagged fruits was sampled at 50, 80 and 120 DAFB, and was subjected to total RNA extraction and RNA-Seq analysis. High-throughput sequencing generated 44.13–59.40 million (M) raw reads from each library (**Table 1**). After raw reads filtered, 42.28-56.18 M clean reads were obtained, with 6.34-8.43 G clean base (**Table 1**). Reads were mapped to the genome sequence of mango cv. ‘Hongxiangya’, and 38.55-51.38 M mapped reads, and 37.02-49.30 M unique mapped reads were generated (**Table 1**). The percentages of mapped reads and unique mapped reads were similar among 18 libraries, with the average of 91.42%, and 87.76%, respectively (**Table 1**). The percentages of error rate of sequencing, Q20, Q30, and GC content among all the libraries were about 0.02%, 98.62%, 95.67%, and 43.52%, respectively (**Table 1**).

**Table 1:** Statistics on the quality and output of the RNA-Seq libraries.

Classification	Maximum	Minimum	Average
Raw Reads	59402606	44133326	50262276
Clean Reads	56176338	42281798	47825796
Clean Base(G)	8.43	6.34	7.17
Mapped reads	51380867	38549444	43724350
% of mapped reads	91.04	91.97	91.42
Unique mapped reads	49298332	37019140	41970161
% of unique mapped reads	88.39	87.14	87.76
Error Rate(%)	0.02	0.02	0.02
Q20(%)	98.73	98.48	98.62
Q30(%)	95.95	95.35	95.67
GC Content(%)	43.83	43.03	43.52
Assembled known genes	29760		
Assembled new transcripts	2623		
DEGs	16239		

The clean reads were assembled into transcripts and compared with the mango genome database (including 34529 genes). Totally, 29760 known genes (86.19% of the total genes) and 2623 new transcripts were obtained (**Table 1**). After screened with the criteria mentioned in Material and Methods section, 16239 differentially expressed genes (DEGs) were identified for further analysis (**Table 1**).

### Validation of DEGs by qPCR

**Figure 2:**
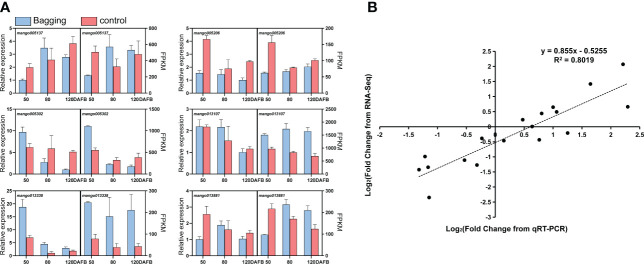
Validation of differentially expressed transcripts by qPCR. **(A)** Gene expression of candidate genes analyzed by qPCR and RNA-seq. Data are presented as the mean ± standard deviation of three biological replicates. **(B)** Correlation analysis based on RNA-seq data and qPCR.

To confirm the accuracy and reliability of the RNA-seq data, 6 DEGs were randomly chosen and analyzed by qPCR. The expression of candidate genes detected by RNA-seq and qPCR showed large consistence (**Figure 2A**), with a significant correlation coefficient of 0.8019 between the two approaches (**Figure 2B**).

### Analysis of DEGs expression trends

**Figure 3:**
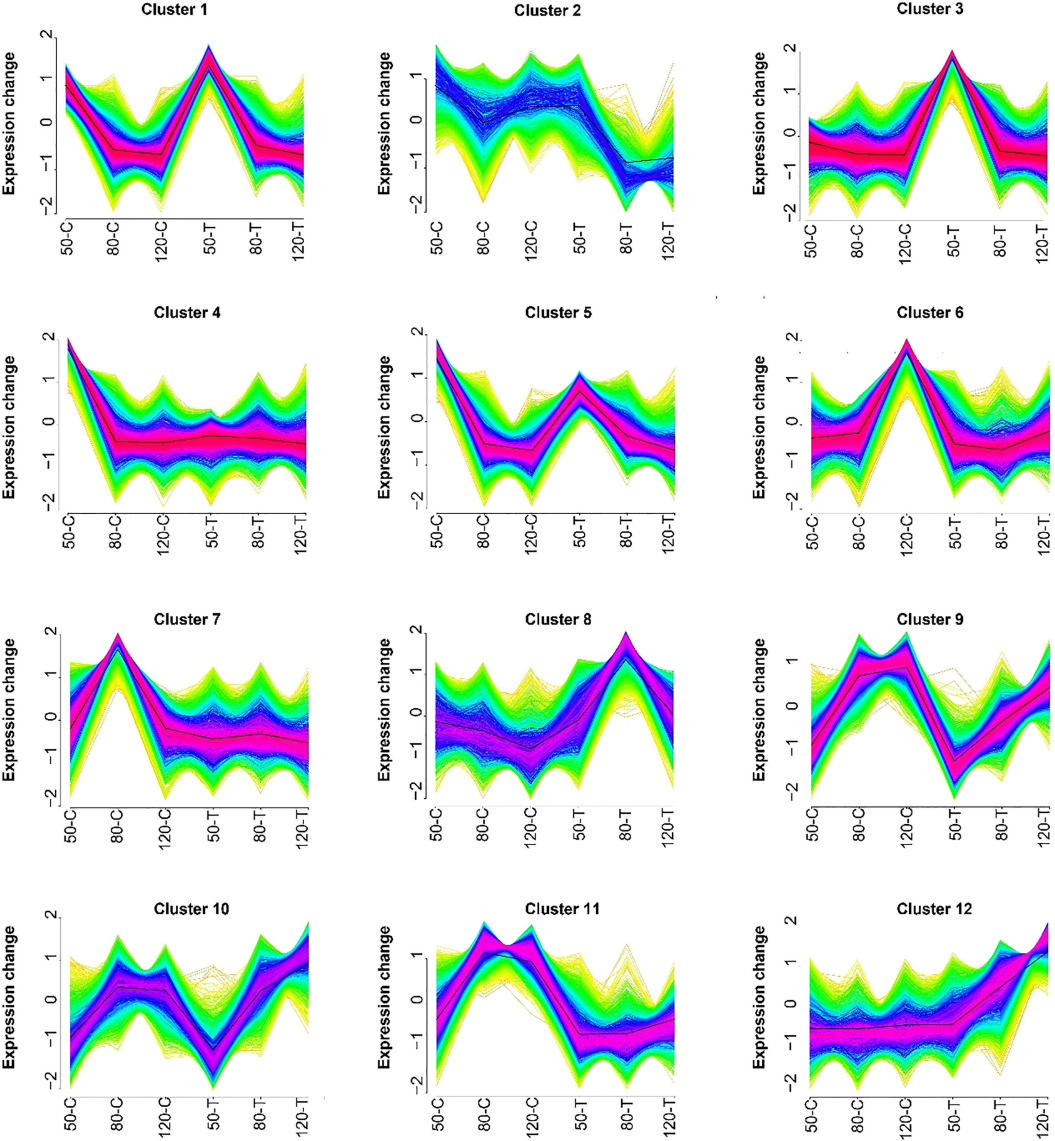
Results of the Mfuzz clustering of differentially expressed transcripts. 50-C, 80-C, and 120-C represent control fruits (natural light grown) sampled at 50, 80, and 120 days after full bloom (DAFB); 50-T, 80-T, and 120-T represent treated fruits (bagged) sampled at 50, 80, and 120 DAFB.

To investigate the effect of natural light on gene expression, all DEGs were analyzed by Mfuzz and grouped into 12 clusters (**Figure 3**). Clusters 2, 3, 4, 6, 7, and 12 showed no clear regular pattern responding to fruit development or light condition (**Figure 3**). Genes from clusters 1 and 5 were down-regulated during development in both natural light grown and bagged fruits, while genes from clusters 9 and 10 were up-regulated during the developing process, so genes from these four clusters were identified as development responsive genes (**Figure 3**). Genes from cluster 8 were highly expressed in bagged fruits, while genes from cluster 11 were highly expressed in natural light grown fruits, so genes from clusters 8 and 11 were identified as negtive and postive light-responsive genes, respectively (**Figure 3**).

### WGCNA revealed flavonoids-related DEGs

**Figure 4:**
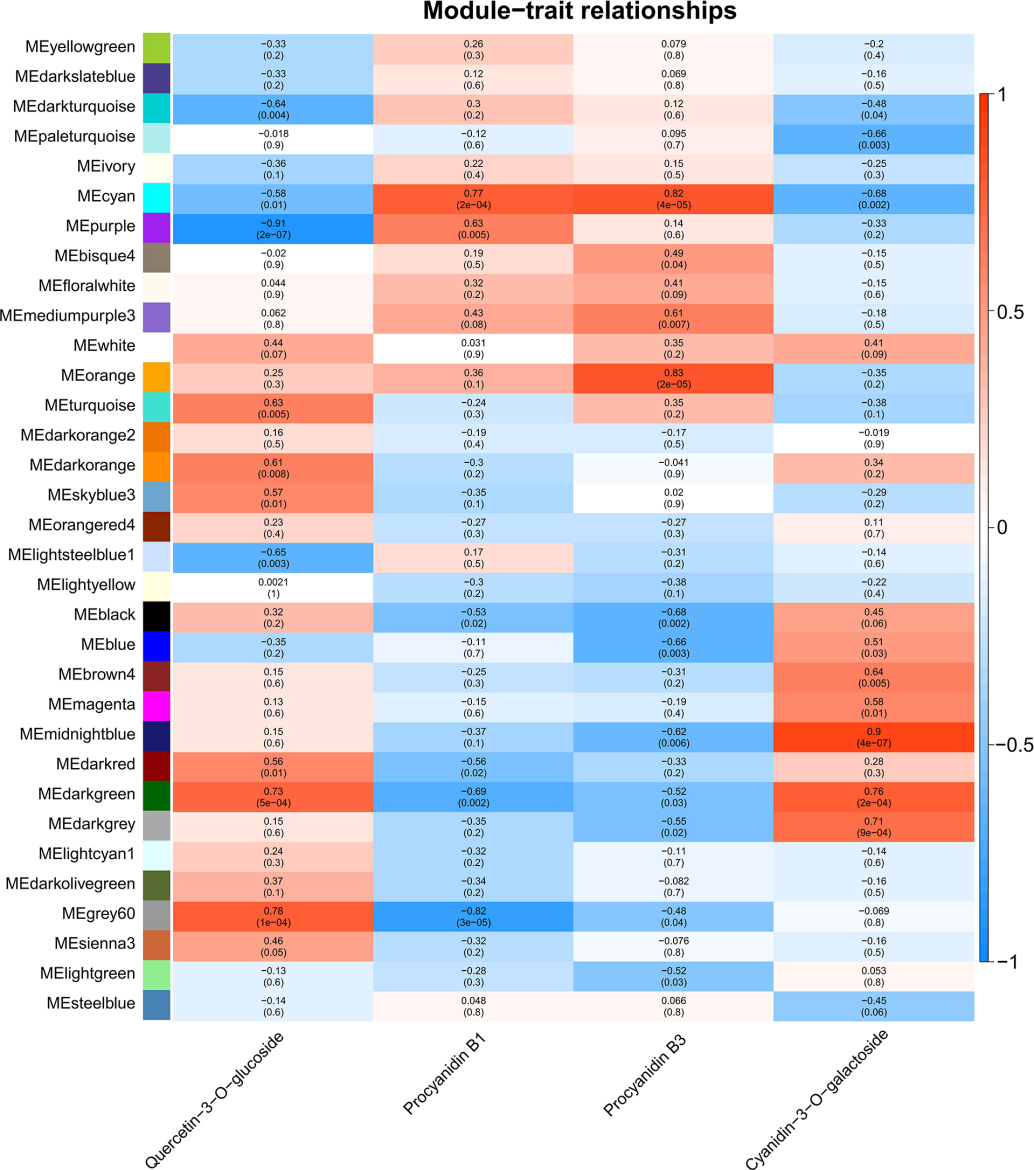
Weighted gene co-expression network analysis (WGCNA) of DEGs identified from transcriptome sequencing. Module-trait correlations and corresponding p-values in parentheses. The left panel shows the 33 modules. The color scale on the right shows the module-trait correlations from -1 (blue) to 1 (red). ‘Quercetin-3-*O*-glucoside’, ‘Procyanidin B1’, ‘Procyanidin B3’ and ‘cyanidin-3-*O*-galactoside’ represent the changes in corresponding substances concentration.

To identify flavonoids biosynthesis-related transcripts, weighted gene co-expression network analysis (WGCNA) was performed, and 33 WGCNA modules were identified (**Figure 4**). Module-trait relations showed that purple module was highly negatively correlated to quercetin-3-*O*-glucoside content (*r* = -0.91, *p* = 2×10^-7^) (**Figure 4**). For proanthocyanidins, grey60 (*r* = -0.82, *p* = 3×10^-5^) showed the highest negative correlation to the concentration of procyanidin B1, and modules cyan (*r* = 0.82, *p* = 4×10^-5^) and orange (*r* = 0.83, *p* = 2×10^-5^) were highly positively correlated to procyanidin B3 content (**Figure 4**). For anthocyanins, module midnightblue exhibited the highest positive correlation to the concentration of cyanidin-3-*O*-galactoside (*r* = 0.9, p = *4*×10^-7^) (**Figure 4**). All in all, genes from purple, grey60, cyan, orange, and midnightblue modules were regarded as candidates regulating natural light-induced flavonoids biosynthesis in mango.

### KEGG analysis of candidate genes revealed by WGCNA

**Figure 5:**
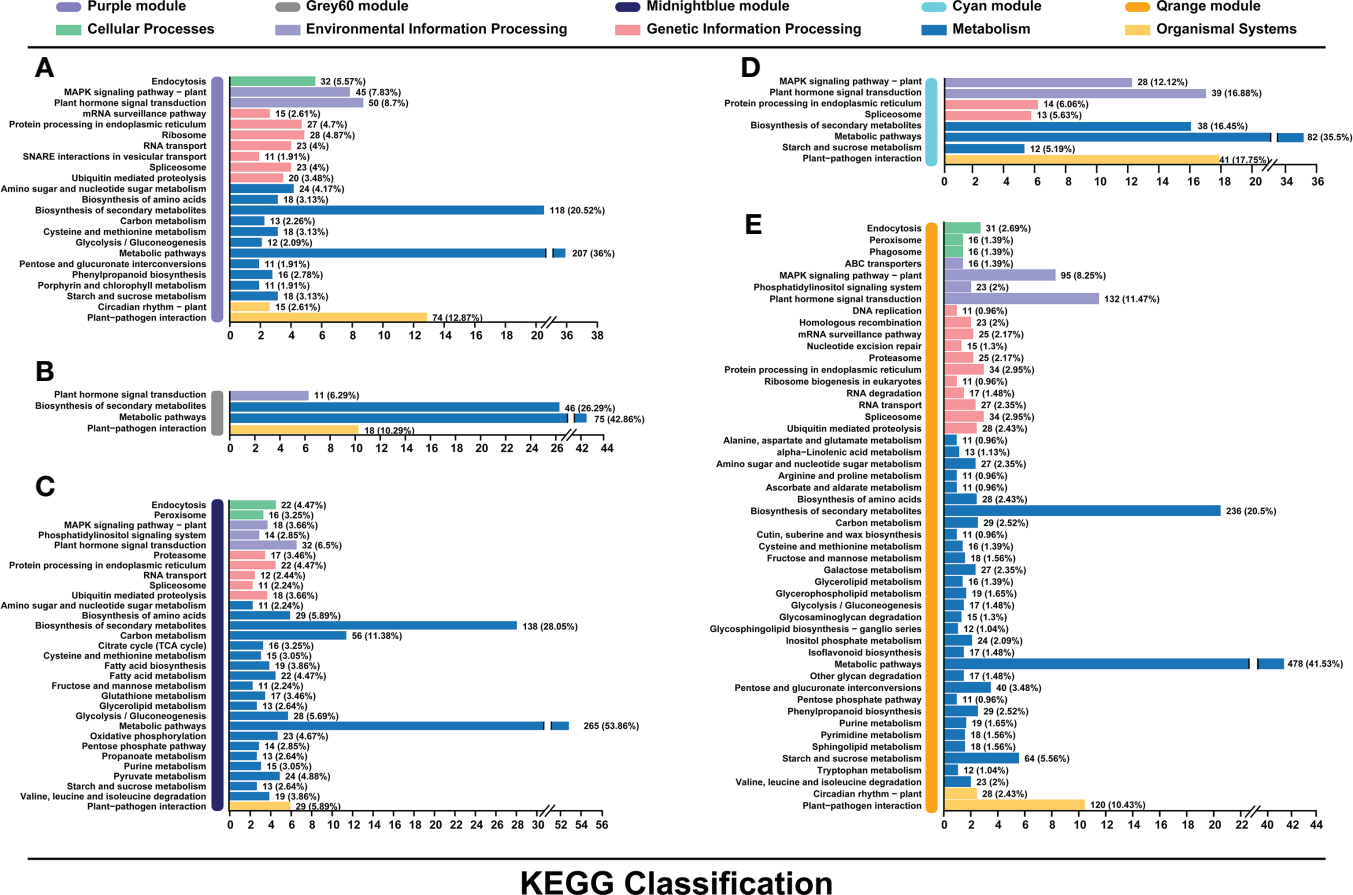
Kyoto Encyclopedia of Genes and Genomes **(KEGG)** pathway enrichment analysis of genes in the purple **(A)**, grey60 **(B)**, midnightblue **(C)**, cyan **(D)**, and orange **(E)** modules associated with the flavonoids biosynthesis.

Candidate genes from purple, darkgreen, grey60, cyan, orange, and midnightblue modules were further performed by Kyoto Encyclopedia of Genes and Genomes (KEGG) analysis. Genes were mainly classified into metabolic pathways (36% in purple, 42.86% in grey60, 35.5% in cyan, 41.53% in orange and 53.86% in midnightblue) and biosynthesis of secondary metabolites (20.52% in purple, 26.29% in grey60, 16.45% in cyan, 20.5% in orange and 28.05% in midnightblue) (**Figure 5**). Except for midnightblue module, plant-pathogen interaction pathway was also enriched by the candidate genes (12.87% in purple, 10.29% in grey60, 17.75% in cyan, and 10.43% in orange) (**Figures 5A, B, D, E**). In addition, plant hormone transduction pathway was enriched in cyan (16.88%) and orange (11.47%) modules (**Figures 5D, E**).

### Flavonoids biosynthetic genes, *MYB*, and *bHLH* revealed by WGCNA

**Figure 6:**
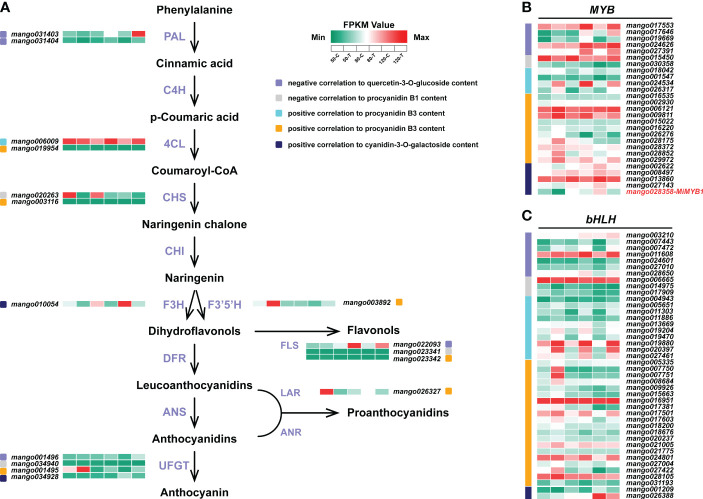
Flavonoids biosynthetic genes **(A)**, *MYB* **(B)**, and *bHLH* **(C)** identified by WGCNA. The color scale from green to red represents the FPKM values from low to high.

In total, 16 structural genes of flavonoid biosynthesis were identified by WGCNA (**Figure 6A**). Two *PALs*, one *FLS*, and one *UFGT* were negatively correlated to flavonol content. One *CHS*, one *FLS*, and one *UFGT* were negatively correlated to procyanidin B1 content. Two *4CLs*, one *CHS*, one *F3’5’H*, one *FLS*, one *LAR*, and one *UFGT* were positively correlated to procyanidin B3 content. One *F3H* and one *UFGT* were positively correlated to anthocyanin content.

For *MYBs*, 15 members were positively correlated to procyanidin B3 content, followed by 5 members negatively correlated to flavonol content, and 5 members positively correlated to anthocyanin content (**Figure 6B**). 2 members were identified to be negatively correlated to procyanidin B1 content. The reported *MYB* controlling anthocyanin biosynthesis in mango, *MiMYB1*, was identified in the midnightblue module, which was positively correlated to anthocyanin content (**Figure 6B**).

For *bHLHs*, a total of 42 members were identified to be related to flavonoids biosynthesis (**Figure 6C**). 30 of them were positively correlated to procyanidin B3 biosynthesis. 7 and 3 *bHLHs* were negatively correlated to flavonol and procyanidin B1 accumulation, respectively (**Figure 6C**). Two members were positively correlated to anthocyanin content (**Figure 6C**).

### Regulatory genes identified by WGCNA

**Figure 7:**
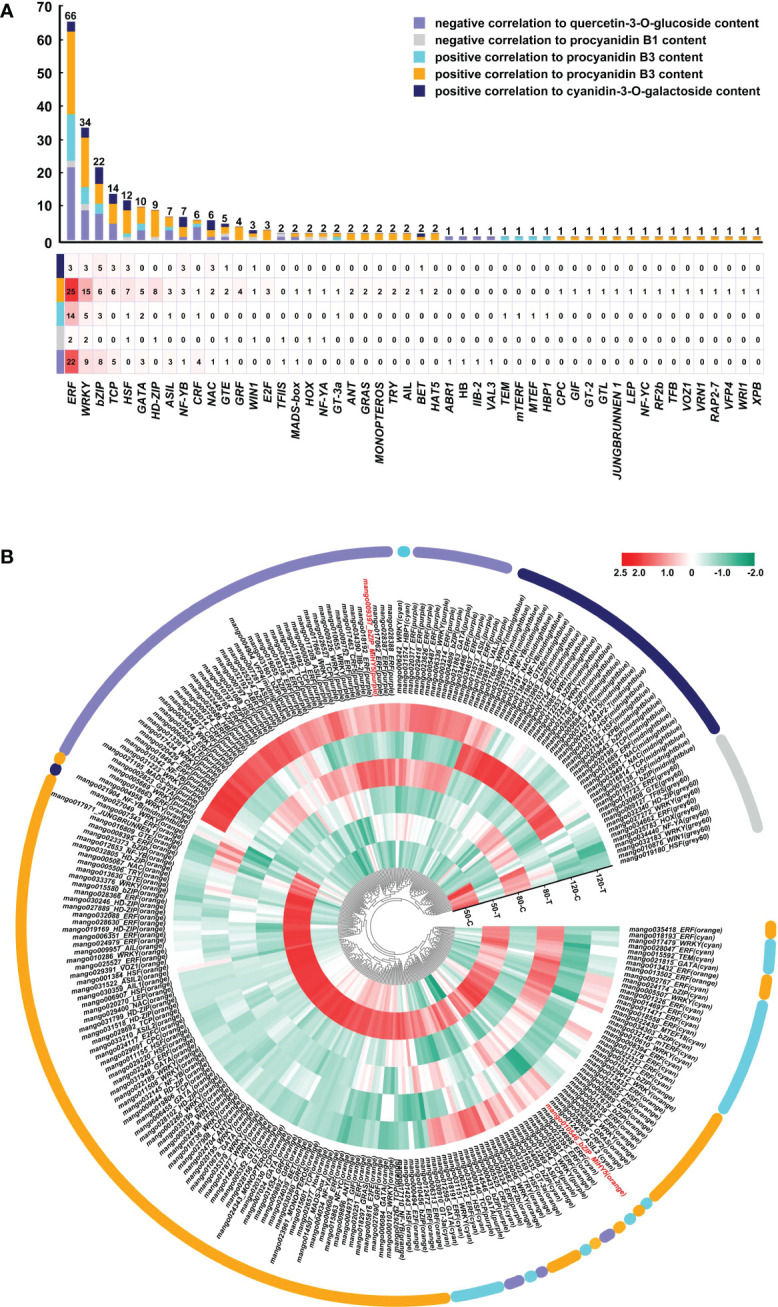
Regulatory genes involved in light-regulated flavonoids biosynthesis. **(A)** Number of regulatory genes in different families. **(B)** Heatmap presenting the expression patterns of different regulatory genes in response to bagging treatment.

A total number of 50 regulatory gene families encoding transcription factors involved in flavonoids biosynthesis were identified (**Figure 7A**). Among them, *ERF* was the most abundant family, with 39 members positively correlated to procyanidin B3 biosynthesis, 22 members negatively correlated to flavonol biosythesis, 3 members positively correlated to anthocyanin biosynthesis, and 2 members negatively correlated to procyanidin B1 biosythesis (**Figure 7A**). *WRKY* and *bZIP* took the second and third place, with 34 and 22 members related to flavonoids biosynthesis, respectively (**Figure 7A**). The rest regulatory genes with more than 10 members identified included *TCP*, *HSF*, and *GATA* (**Figure 7A**). **Figure 7B** showed that most genes were positively correlated to procyanidin B3 biosynthesis (from cyan and orange modules), with a stunning high expression in the bagged fruit at 50 DAFB. Genes negtively correlated to flavonol were also highly enriched, which was general highly expressed in bagged fruit at 80 and 120 DAFB. Genes positively correlated to anthocyanin biosynthesis from midnightblue showed an obvious up-regulation expression in the control fruit at 120 DAFB. Surprisingly, two members of *MiHY5*, which encode the most important transcription factor in light signal, were identified to be negatively correlated to flavonol biosynthesis, and positively correlated to procyanidin B3 biosynthesis, respectively (**Figure 7B**).

## Discussion

Among all the environmental factors, light plays an essential role in regulating flavonoids biosynthesis (Wen et al., 2020). Our results showed that sunlight exposure increased the accumulation of flavonols and anthocyanins (**Figure 1**) (Shi et al., 2021), which was similar with the previous study (Niu et al., 2010; Qian et al., 2013; Ma et al., 2021). However, bagged fruit peel showed higher level of proanthocyanidins, which was opposite from the other studies, in which bagging treatment usually inhibits the proanthocyanidins accumulation in fruits (Scafidi et al., 2013; Wang et al., 2015). So it is interesting to investigate the molecular mechanism of light-regulated accumulation of different flavonoids compositions in mango.

Among all the 16 flavonoids biosynthetic genes identified by WGCNA, 5 genes were induced by light while 11 genes were repressed by light (**Figure 6A**), indicating the competition of different flavonoids compositions biosynthesis is through the enzymes of flavonoids pathway. As flavonoids are induced by light in most cases, numerous studies reported that flavonoids biosynthetic genes are also up-regulated by light (Bai et al., 2017; Qian et al., 2017; Qian et al., 2019). However, almost all the flavonoids biosynthetic genes exist in gene family, so some gene members could also be inhibited by light (Qian et al., 2013). All these results suggested that under dark condition, some flavonoids structural genes should also be highly expressed to ensure the necessary accumulation of flavonoids components, which are helpful for the fruits against various biotic and abiotic stresses during development. In addition, *LAR* (*mango026327*) expression was significantly induced by bagging treatment (**Figure 6A**), suggesting *LAR* is the key gene responsible for the proanthocyanidins biosynthesis in the bagged fruit peel. As the enzyme catalyzing the last step of the proanthocyanidins biosynthesis, *LAR* expression is usually positively correlated to the concentration of proanthocyanidins in strawberry (Schaart et al., 2013), apple (Wang et al., 2017), and grape (Lacampagne et al., 2010).

MYB and bHLH are the two most essential transcription factors controlling flavonoids biosynthesis, which form a complex and bind to the promoter region of structural genes through MYB to regulate the expression of structural genes (Xu et al., 2015). Apart from the activator MYBs, which promote the biosynthesis of flavonols (Wang et al., 2017; Premathilake et al., 2020), proanthocaynidins (Schaart et al., 2013; Wang et al., 2017), and anthocyanins (Kobayashi et al., 2004; Takos et al., 2006; Feng et al., 2010), repressor MYBs have also been reported. In pear, PpMYB140 could inhibit anthocyanins biosynthesis by repressing the expression of anthocyanins biosynthetic genes, as well as competing with the activator PpMYB114 to interact with bHLH3 (Ni et al., 2021). In poplar, overexpression of the repressors *MYB165* and *MYB194* could tremendously reduce the accumulation of anthocyanins and proanthocyanidins (Ma et al., 2018). Similar to MYB, the regulation of flavonoids by bHLH is also mediated by both activators and repressors (Xie et al., 2012; Zhao et al., 2018; Tao et al., 2020; Zhao et al., 2020). In the present study, 20 activator *MYBs*, 7 repressor *MYBs*, 32 activator *bHLHs* and 10 repressor *bHLHs* were identified to contribute to the regulation of flavonoids biosynthesis (**Figures 6B, C**), indicating the activation through MYB and bHLH is dominating in regulating flavonoids accumulation when compared with repression regulation. However, repressors are necessary to develop a fine-tuning regulatory loop to balance the flavonoids biosynthesis and prevent from excess flavonoids accumulation.

Among the other transcription factors, ERF, WRKY, and bZIP showed the highest number of family members identified by WGCNA, including both activators and repressors (**Figure 7A**). Especially for ERF, 22 members were negatively correlated to flavonols concentration (**Figure 7A**), indicating ERF plays a crucial role in the negative regulation of flavonols biosynthesis. ERF and WRKY have been widely reported to regulate flavonoids biosynthesis in fruits. In pear, Pp4ERF24 and Pp12ERF96 could interact with PpMYB114 and enhance the interaction between PpMYB114 and PpbHLH3 to promote the anthocyanin accumulation by blue light (Ni et al., 2019), while PpERF105 could inhibit the anthocyanin accumulation under ethylene treatment by inducing the expression of repressor *PpMYB140* (Ni et al., 2021). WRKY could also promote or inhibit flavonoids accumulation by interacting with activator MYB or bHLH (An et al., 2019; Li et al., 2020), promoting the expression of activator *MYB* (Hu et al., 2020; Alabd et al., 2022), repressing the expression of flavonoids biosynthetic and regulatory genes (Mao et al., 2021), or interacting with the repressor MYB (Mao et al., 2021). Interestingly in apple, the light-induced anthocyanin accumulation is regulated through a MdWRKY1–MdLNC499–MdERF109 transcriptional cascade (Ma H. et al., 2021). MdWRKY1 could induce the expression of a long noncoding RNA, *MdLNC499*, which subsequently promotes the expression of *MdERF109*, and MdERF109 promotes the transcription of anthocyanin-related genes and the anthocyanins accumulation [64]. Among the bZIP transcription factors, HY5 and its homolog HYH are the most important transcription factors in the light transduction pathway and regulate photomorphogenesis such as flavonoids accumulation in plant through activating the expression of flavonoids-related genes (Holm et al., 2002; Zhang et al., 2011; Zhao et al., 2022). Recently, it has been reported that after the rapid induction of HY5 transcription by UV-B light, HY5 could bind to its own promoter to inhibit expression, which forms an autoregulatory negative feedback loop to balance *HY5* transcription (Yang et al., 2022). In the current study, two *HY5s* were identified by WGCNA, and their expression was repressed by light (**Figure 7B**). The possible reasons could be: the *HY5* expression in un-bagged fruit peel undergoes diurnal rhythm, and the expression at the sampling time (10 am in the morning) did not reach the peak expression level in the day; or after the quick response of light, *HY5* expression was inhibited by itself or other transcription factors to prevent excess accumulation. All these results suggest ERF, WRKY, and bZIP play an essential role in light-regulated flavonoids accumulation in mango peel, and the regulation mechanism is divers and complex, which combines both positive and negative regulation.

## Data Availability

The datasets presented in this study can be found in online repositories. The names of the repository/repositories and accession number(s) can be found in the article/**Supplementary Material**.

## Conclusions

Compared with un-bagged mango fruit peel with dark-red coloration, the bagged fruit peel showed a light-yellow coloration, with relatively higher L*, lower a*, and higher b* values. Through RNA-seq, 16239 DEGs were identified and grouped into 12 clusters by Mfuzz analysis, and clusters 8 and 11 were regarded as light-responsive clusters. Through WGCNA analysis, genes from purple, grey60, cyan, orange, and midnightblue modules were involved in light-regulated flavonoids biosynthesis in mango, and most genes were classified into metabolic pathways and biosynthesis of secondary metabolites by KEGG analysis. In addition, key flavonoids biosynthetic genes, as well as activator and repressor TFs including MYB, bHLH, ERF, WRKY and bZIP regulating flavonoids accumulation under bagging treatment were also identified. Our results provide a comprehensive analysis and broad view of the light-regulated flavonoids biosynthesis in mango.
